# BlingLife^®^-Marigold Extract Alleviates Blue Light-Induced Retinal Mitochondria Oxidative Stress and Senescence by Activating NRF2/HO-1 Signaling

**DOI:** 10.4014/jmb.2411.11037

**Published:** 2025-02-24

**Authors:** Zixiu Zhou, Sizhen Li, Qingsong Yang, Pengjie Zheng, Kexin Xie

**Affiliations:** 1 Nanjing Tongren Hospital, School of Medicine, Southeast University, Nanjing, Jiangsu 211102, P.R. China; 2 State Key Laboratory of Pharmaceutical Biotechnology, School of life Sciences, Nanjing University, Nanjing, Jiangsu 210023, P.R. China

**Keywords:** Retinal damage, blue light, reactive oxygen species, meso-zeaxanthin, NRF2/HO-1 signaling

## Abstract

Blue light poses a risk of retinal damage with excessive exposure. BlingLife^®^-marigold extract (BLM) is an alcohol extract of magrigold, which contains abundant lutein, zeaxanthin and meso-zeaxanthin. This study aimed to explore the role and potential mechanisms of BLM in blue light-induced retinal damage both in vivo and in vitro. Rats or human retinal pigment epithelial cell line (ARPE-19) were exposed to blue LED light with or without BLM treatment. The retinal morphology changes of rat were evaluated by H&E staining. Mitochondrial morphology was examined by using a transmission electron microscope. Besides, mitochondria oxidative stress was evaluated by detecting mitochondrial membrane potential, ROS, MDA and SOD levels. By measuring γH2AX expression and performing SA-β-galactosidase (gal) staining, cell senescence was assessed. Additionally, cell cycle was detected using flow cytometry. Western blot was employed to examine the expression of NRF2 and HO-1. Results indicated that BLM could protect against blue light-induced damage of rat retinal tissues and ARPE-19 cells, as evidenced by the improved histopathological changes, alleviated mitochondria oxidative stress and attenuated senescence of tissues and cells. More importantly, BLM activated NRF2/HO-1 signaling, and addition of NRF2 inhibitor ML385 significantly blocked the protective effects of BLM on ARPE-19 cells exposed to blue light. In conclusion, BLM can provide an effective protection against blue light-induced retinal damage at least partly by activating NRF2/HO-1 signaling, suggesting that BLM may be useful for the prevention of blue light-induced retinal injury.

## Introduction

In recent years, the widespread use of electronic devices, including smartphones, digital displays, and computer screens, the exposure time of people's eyes to blue light has increased significantly [1]. As high-energy visible, blue light (from 450 to 495 nm) penetrates the lens to reach the retina more strongly than ultraviolet light [2, 3]. As demonstrated, blue light damages retina and lens, leading to several ocular diseases [4-6]. The correlation between blue light and age-related macular degeneration (AMD), a common blinding disease that results in permanent vision impairment and loss among the elderly population, has also been disclosed in epidemiological studies [7, 8]. Therefore, there is an urgent and significant need to develop agents that can prevent and mitigate blue light-triggered optical damage. As reported, damage to retinal cells induced by blue light is linked to the excessively generated ROS, mitochondrial dysfunction, DNA damage, and senescence [9-11]. BlingLife^®^-marigold extract (BLM) is an alcohol extract of marigold (*Tagetes erecta* L.), which contains at least 85% by weight of total xanthophylls, of which at least 80% by weight being lutein, the remaining 15% by weight being zeaxanthin isomers, namely zeaxanthin, and meso-zeaxanthin. Marigold, also called *Calendula officinalis* L., belongs to the Calendula family of Asteraceae with yellow or orange flowers that are broken up and used to make herbal preparations [12]. Marigold has been proven to be one of the largest commercial sources of pure lutein and zeaxanthin [13]. Lutein is an oxygen-containing carotenoid pigment that, together with zeaxanthin, forms the macular pigment, which accumulates mainly in the retina and lens [13]. Lutein and zeaxanthin not only have a strong antioxidant effect, but also have a strong absorption of short-wavelength light involving blue light. A previous study has reported that supplementing carotenoids containing lutein and zeaxanthin (10:1) protects macular pigments against oxidative damage in Swiss albino rats [14]. Meso-zeaxanthin is the other one of the three xanthophyll carotenoids found in the *macula lutea*. It is primarily derived from the endogenous conversion of lutein in the retinal pigment epithelium within the eye [15, 16]. The predominant carotenoids at the epicentre, midperiphery, and periphery of the macula are meso-zeaxanthin, zeaxanthin, and lutein, respectively [17]. Therefore, the macula might be expected to derive benefit from the greater protection afforded by meso-zeaxanthin, and zeaxanthin compared with lutein. In the past twenty years, intervention trials have investigated the impacts of lutein, zeaxanthin and meso-zeaxanthin on human health [18]. As one of the best commercial sources of macular pigment, the effects of marigold extract on retinal damage triggered by blue light and the possible mechanism remains unclear.

In this work, the main active ingredients of BLM are identified to be lutein, zeaxanthin, and meso-zeaxanthin, by mass spectrometry. Then, the impacts of BLM on blue light-triggered damage of rat retina and human retinal pigment epithelial cells (ARPE-19) were explored. Further, the potential mechanisms of BLM on nuclear factor E2-related factor 2 (NRF2)/heme oxygenase-1 (HO-1) signaling were studied.

## Materials and Methods

### Animals

Male SD rats (200-220 g) obtained from Shang hai Bikai Keyi Biotechnology Co., LTD. were raised in the SPF environment under a 12 h light/dark regimen with abundant supplement of food and water. Animals were conventionally adaptively bred for one week. Animals were randomized into four groups (18 rats/group): control, model (blue light irradiation), low (blue light irradiation and intragastric administration of 7.875 mg/kg BLM group), and high (blue light irradiation and intragastric administration of 94.5 mg/kg BLM group) groups. In drug-treated groups, rats were given the corresponding dose of BLM once a day for 45 consecutive days. Afterwards, rats in all other groups aside from the control group were treated with blue LED light continually (wavelength: 480 nm; Opple Lighting Co., Ltd., China) for 3 h. After acclimatization to the dark for 72 h, a 200 mg/kg intraperitoneal injection of sodium pentobarbital was used to euthanize the rats. Abdominal aorta blood and eyeballs were collected for following experiments. Animal procedures were approved by institutional ethics committee board of Zhaofenghua Biotechnology Co., Ltd., (approval number: IACUC-20230510). All procedures were in accordance with the Guide for the Care and Use of Laboratory Animals (National Institutes of Health).

### Pathological Examination

The eyes were sealed in 4% formaldehyde for a whole day and then dehydrated and embedded in paraffin. The 5 μm thick deparaffinized retinal tissues were rehydrated in graded ethanol. After staining with hematoxylin and eosin (H&E), samples were evaluated under a light microscope (Olympus, Japan) for observation.

### Culturing ARPE-19 Cells

ARPE-19 cells (Procell, China) were maintained in DMEM (Gibco, USA) containing 10% FBS. The conditions of incubator were set as 37°C, 95% air and 5% CO_2_. The abdominal aorta blood of rats in the High group was taken and serum was acquired by centrifugation. A 0.22-micron bacteria-retentive filter was used to sterilize the serum, and it was inactivated for 45 min at 56°C. ARPE-19 cells were cultivated in DMEM containing 5%, 10%, 15%, 20%and 25% contained-BLM serum for 24 h.

### Blue Light Irradiation

When incubated for 20 h in contained-BLM serum, cells were irradiated with blue light (100% blue illuminometer, 470 nm) for 4 h. The unirradiated ARPE-19 cells cultured in 10% FBS were used as a control.

### Detection of Cell Viability

RPE-19 cells were exposed to 10 μl CCK-8 reagent for 2 h. A microplate reader (Bio-Rad, USA) was used to analyze the viability of the cells at the absorbance of 450 nm.

### Mitochondrial Morphological Observation

After the indicated treatment, eyes without lens and cornea and ARPE-19 cells were placed in 2.5% glutaraldehyde, immersed in 1% osmium tetroxide, and embedded in epoxy resin. Lead citrate and uranyl acetate were used to stain the ultrathin slices. Images were photographed with the application of a transmission electron microscope.

### Detection of ROS Level

The sections (4 μm thickness) of retinal tissues or or ARPE-19 cells were dyed by 5 μM dihydroethidium (DHE) for 30 min avoid light. After three rinses in PBS, the fluorescence intensity was acquired with the adoption of a fluorescence microscope (Olympus).

### Mitochondrial Membrane Potential (MMP)

5,5',6,6'-tetrachloro1,1',3,3'-tetramethylbenzimidazolylcarbocyanine iodide (JC-1) is an ideal fluorescent probe used in the detection of MMP. For *in vivo* studies, we resuspended the purified mitochondria and mix them with JC-1 solution (Solarbio, China). The cultured ARPE-19 cells were treated with 10 μM JC-1 dye (Beyotime, China) for 20 min away from light. A fluorescence microscope was employed to analyze the fluorescence intensity.

### Measurement of Oxidative Stress Markers

Retinal tissues were homogenized with PBS and then the supernatant was extracted by centrifuging. MDA and SOD levels in homogenate supernatant and ARPE-19 cells were examined through the commercial available kits (Mlbio, China). The OD value was taken under a microplate reader (Bio-Rad).

### SA-β-Galactosidase (gal) Staining Assay

The activity of SA-β-gal in frozen retinal tissues sections (4 μm) and ARPE-19 cells was examined to evaluate tissue senescence and cellular senescence utilizing a kit (Beyotime). Photos were gathered randomly using a light microscope. Image J software was adopted for calculating the proportion of SA-β-gal positive areas/cells.

### Immunohistochemical Analysis

The 5 μm thick deparaffinized retinal tissues were exposed to citrate buffer, which were then permeated with 5% BSA and 0.1% Triton X-100 for 1 h. Subsequently, the sectioned tissues were probed with an anti-γH2AX antibody at 4°C overnight, after which a one-hour incubation period was spent using a secondary antibody (Abbkine, China). The slides were dyed with DAB and haematoxylin. The images were obtained by virtue of a light microscope. The proportion of γH2AX positive areas were analyzed through Image J software.

### Immunofluorescence Staining

After being placed in 4% paraformaldehyde, ARPE-19 cells were sequentially exposed to 0.2% Triton X-100 and 5% BSA for 1 h. The primary antibody against γH2AX (Proteintech, USA) was probed with the cells at 4°C overnight. Afterwards, cells were probed with Alexa 488-linked second antibody, and the culture was continued for 1.5 h. Nuclei were counterstained with DAPI and avoid exposure to light. A fluorescence microscope was applied for observing the images.

### Cell Cycle Assay

ARPE-19 cells were placed in 70% ethanol at 4°C overnight. Afterwards, cells were labeled with 500 μl PI (Solarbio) staining buffer containing 100 ug/ml RNase A (Sigma‐Aldrich) avoid light. A flow cytometry (BD Biosciences, USA) was adopted for measuring cell cycle.

### Western Blot

The total proteins in retinal tissues or ARPE-19 cells were extracted employing RIPA lysis buffer. The quantitative determination of proteins was implemented with a Bradford assay. Proteins were loaded on SDS-PAGE and transferred to nitrocellulose filters. These blots were sealed with 5% BSA for 1.5h, followed by incubation with primary antibodies (Proteintech). Afterwards, the HRP-labeled second antibody (Abbkine) was added, and the membranes were probed with it for extra 1 h. Blots were observed using the ECL detection reagent. Signal intensity was analyzed by virtue of ImageJ software.

### Statistical Analyses

GraphPad 8.0 software was applied for data analysis. Data were shown as mean ± standard deviation. Tukey's test was performed after a one-way ANOVA was employed to perform statistical calculations on the data. The significance was set at *p* < 0.05 in statistics.

## Results

### BLM Improves the Histopathological Changes of Rat Retinal Tissues Exposed to Blue Light

**Fig. 1:**
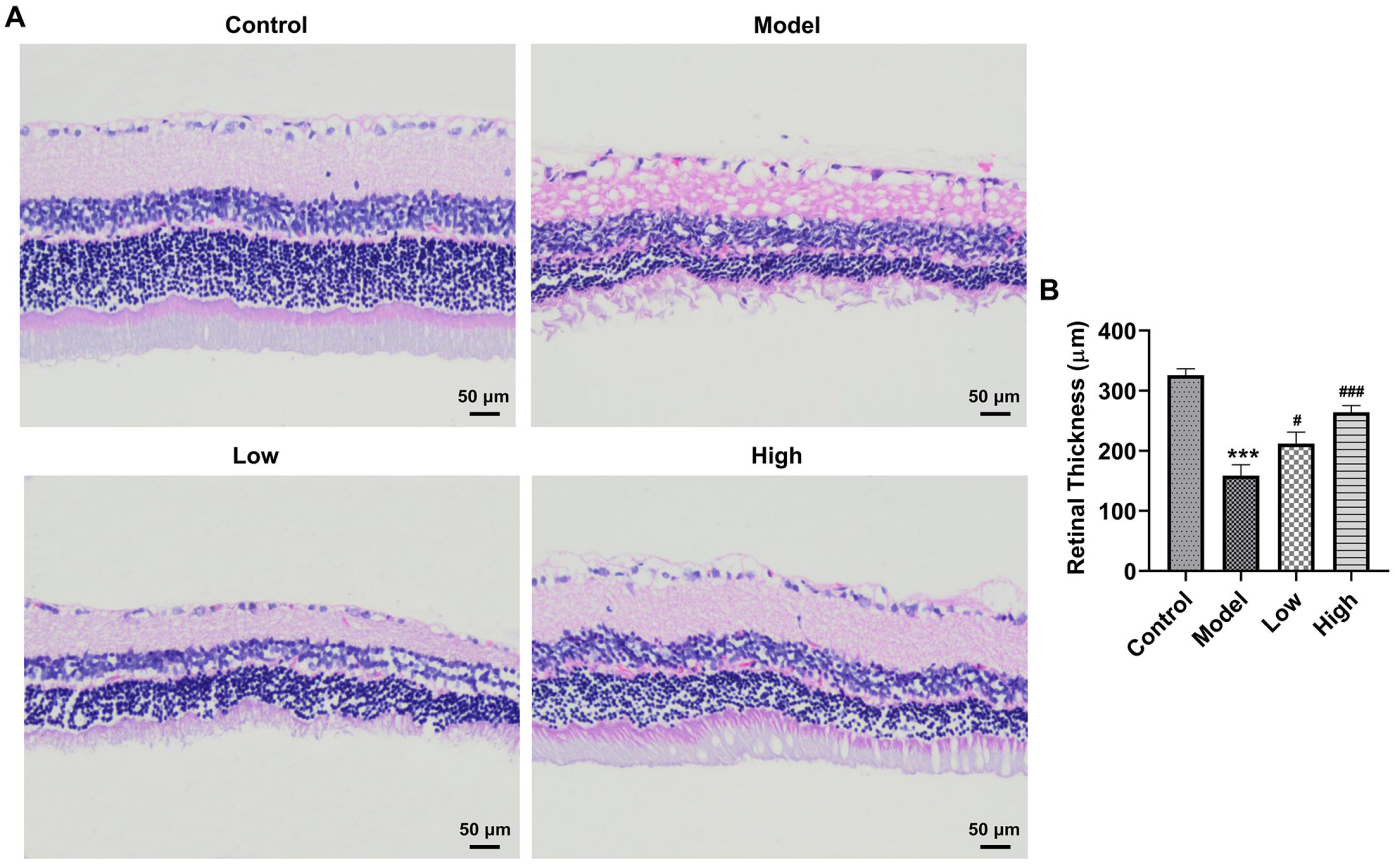
BLM alleviated the histopathological changes of rat retinal tissues exposed to blue light. (**A**) Evaluation of retinal morphology using H&E staining. (**B**) The retinal thickness in H&E staining was quantified. ***p < 0.001 vs. Control group; ^#^*p* < 0.05, ^###^*p* < 0.001 vs. Model group.

Firstly, the retinal morphology changes of rats were evaluated by H&E staining. Contrast to the control group, the blue light-exposed group displayed a significant retinal structure disorder, blurred retinal layer demarcation, and massive photoreceptor loss (Fig. 1A). It could be found that the retinal thickness was notably decreased in blue light-stimulated group relative to the Control group (Fig. 1B). Conversely, the abovementioned histopathological changes treated with blue light were remarkably improved by BLM pretreatment. These data suggest that BLM improves the histopathological changes of rat retinal tissues exposed to blue light.

### BLM Alleviates Mitochondria Oxidative Stress of Rat Retinal Tissues Exposed to Blue Light

**Fig. 2:**
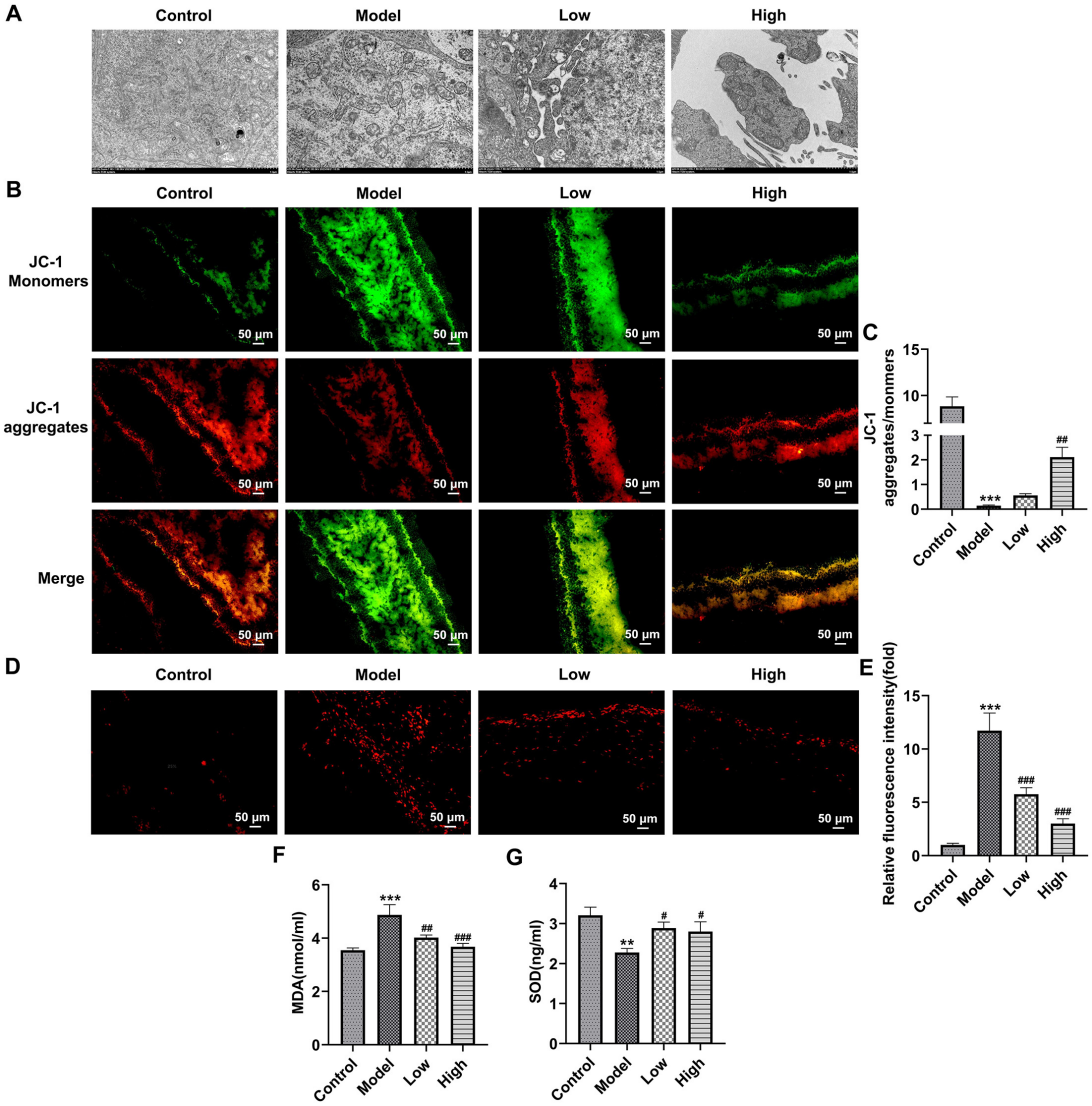
BLM alleviates the mitochondria oxidative stress and oxidative stress of rat retinal tissues exposed to blue light. (**A**) Mitochondrial morphology was examined by a transmission electron microscope. (**B**) Mitochondrial membrane potential was analyzed using JC-1 staining. (**C**) Calculation of the ratio of JC-1 aggregates to JC-1 monomers. (**D**) Detection of ROS using DHE staining. (**E**) Quantification of DHE fluorescence intensity. Measurement of (**F**) MDA and (**G**) SOD levels using the corresponding commercial available kits. ***p < 0.001 vs. Control group; ^#^*p* < 0.05, ^###^*p* < 0.001 vs. Model group.

The mitochondrial morphology was examined by a transmission electron microscope. As exhibited in Fig. 2A, after blue light exposure, mitochondrial damage in retinal tissue was serious, mitochondrial vacuole and ridge breakage appeared. After intervention with BLM, mitochondrial ridge breakage in retina was reduced, and more intact mitochondria were observed in the high-dose BLM group. Additionally, blue light irradiation markedly elevated the level of JC-1 monomers and declined the level of JC-1 aggregates in mitochondria of retina, leading to the reduced ratio of JC-1 aggregates to JC-1 monomers (Fig. 2B and 2C). Following BLM treatment, the reduction in ratio of JC-1 aggregates to JC-1 monomers induced by blue light was increased, especially in the High group, indicating that BLM elevated MMP in blue light-exposed retina. Besides, blue light stimulation notably enhanced the DHE fluorescence intensity, elevated MDA level and decreased SOD activity in retinal tissues as compared to the Control group, which were significantly restored after BLM pretreatment (Fig. 2D-2G). The above results indicate that BLM alleviates the mitochondria oxidative stress of rat retinal tissues exposed to blue light.

### BLM Attenuates the Senescence of Rat Retinal Tissues Exposed to Blue Light

**Fig. 3:**
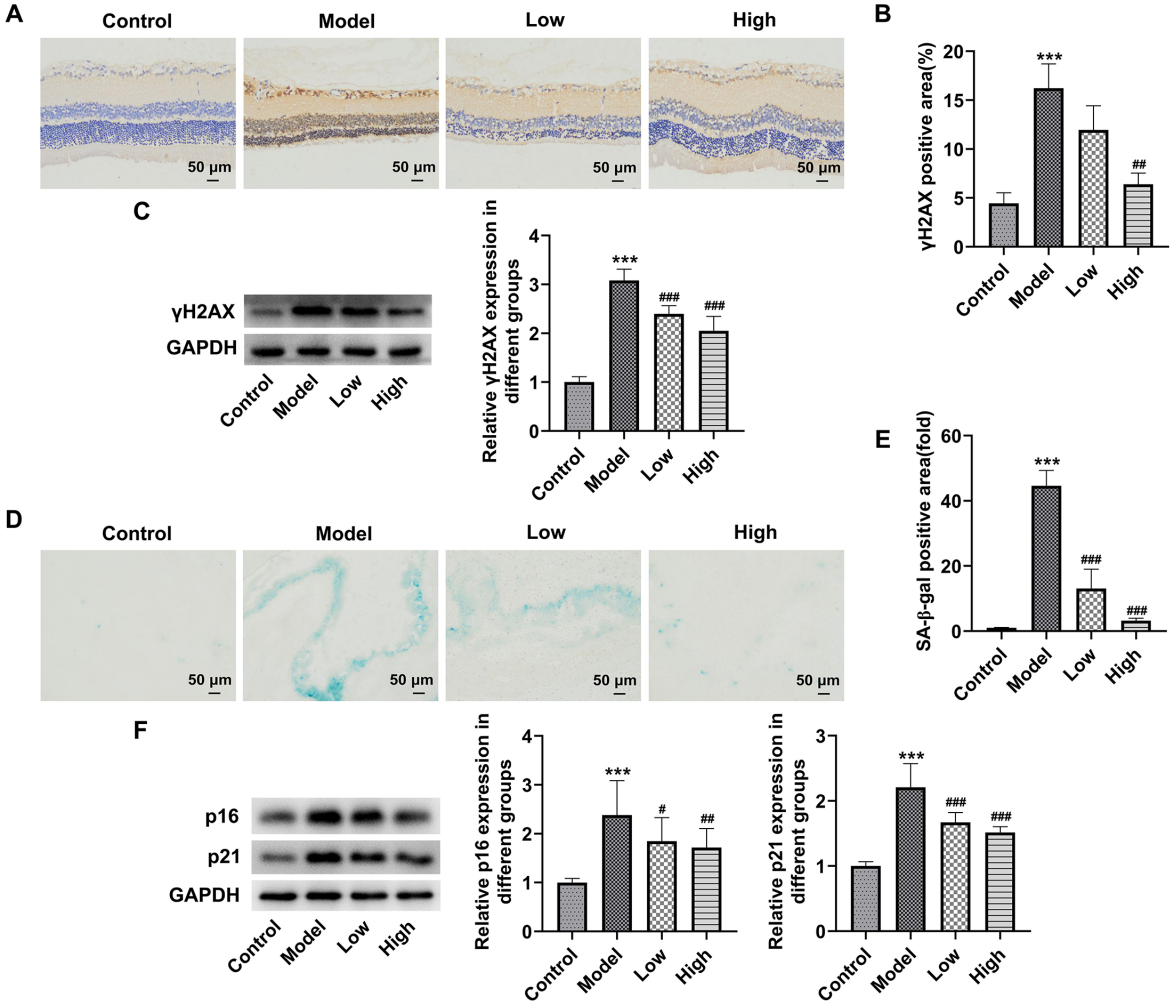
BLM attenuated the senescence of rat retinal tissues exposed to blue light. (**A**) Immunohistochemical analysis was used to analyze γH2AX expression in retinal tissues. (**B**) Quantification of γH2AX positive area. (**C**) western blot assay was employed to measure γH2AX expression in retinal tissues. (**D**) Detection of senescence using SA-β-gal staining. (**E**) Quantification of γH2AX positive area. (**F**) p16 and p21 expression was examined by western blot. ***p < 0.001 vs. Control group; ^#^*p* < 0.05, ^##^*p* < 0.01, ^###^*p* < 0.001 vs. Model group.

Results of immunohistochemical analysis and western blot assay exhibited in Fig. 3A-3C revealed that γH2AX expression was conspicuously upregulated in retinal tissues of rats in the Model group. By contrast, BLM administration attenuated the impact of blue light exposure on γH2AX level in retinal tissues. Moreover, as illustrated in Fig. 3D-3F, as comparison to the Control group, more than 40% area of retinal tissues became senescent after blue light irradiation, accompanied by the upregulated p16 and p21 expression. However, BLM pretreatment reduced the ratio of SA-β-gal positive area and downregulated p16 and p21 levels. These data provide evidence that BLM attenuates the senescence of rat retinal tissues exposed to blue light.

### BLM Upregulates NRF2/HO-1 Pathway in Rat Retinal Tissues and ARPE-19 Cells Exposed to Blue Light

**Fig. 4:**
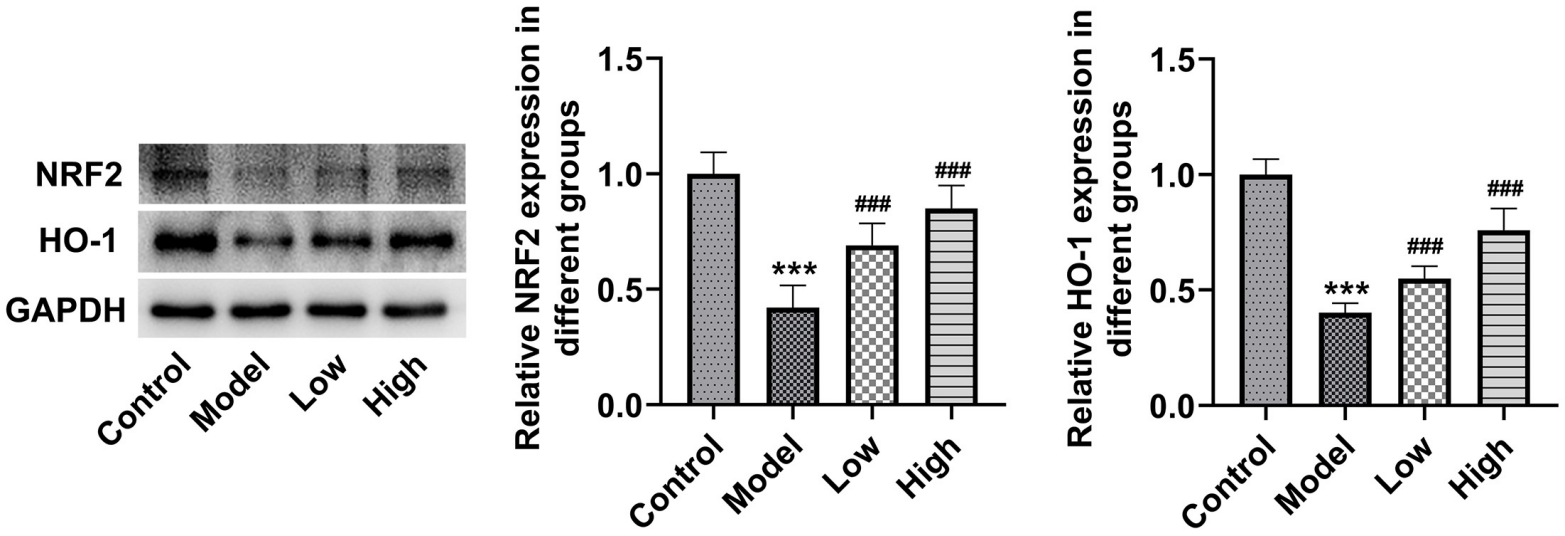
BLM activates NRF2/HO-1 signaling in rat retinal tissues exposed to blue light. Western blot assay was used to examine NRF2 and HO-1 expression in rat retinal tissues. ***p < 0.001 vs. control group; ^###^*p* < 0.001 vs. model group.

**Fig. 5:**
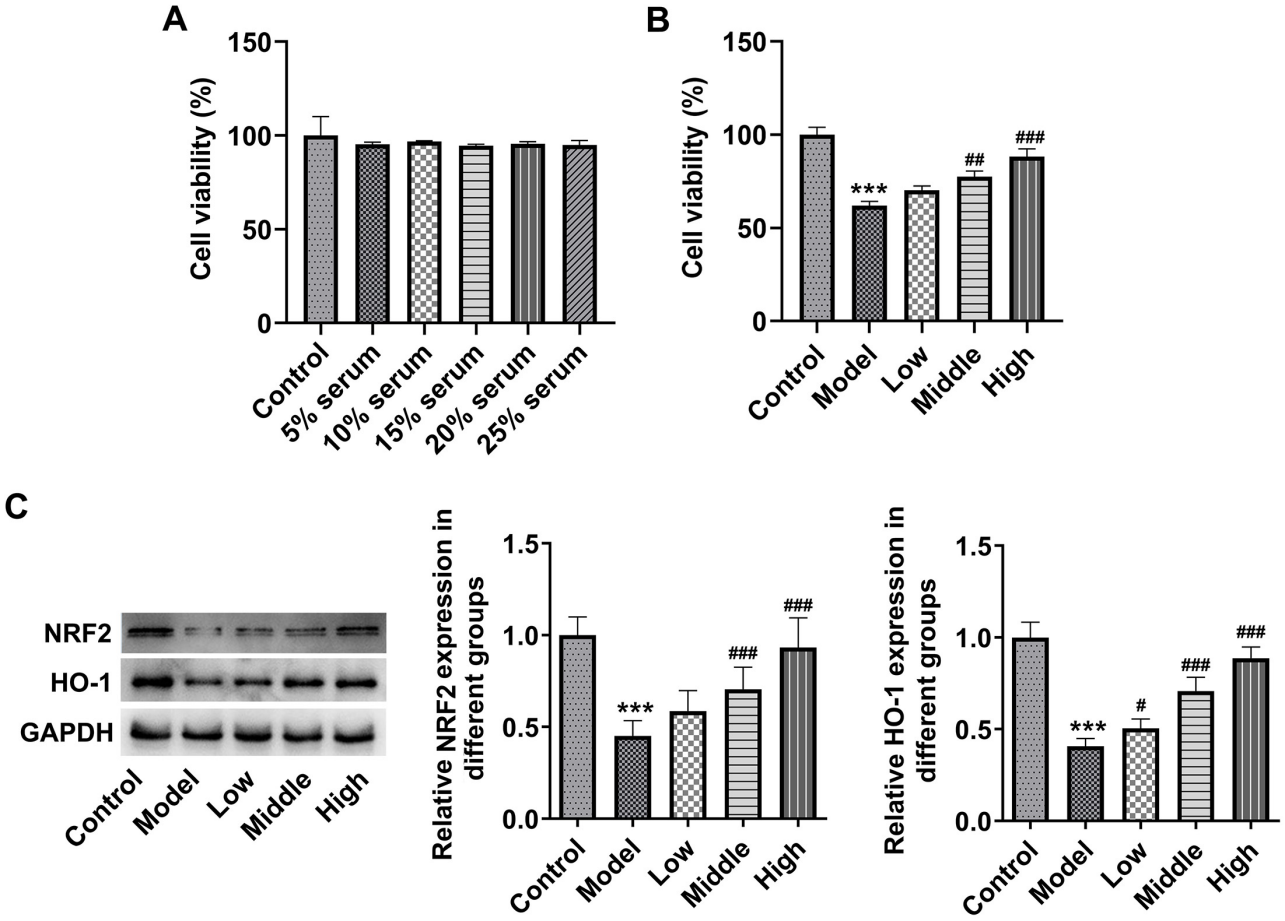
BLM activates NRF2/HO-1 signaling in ARPE-19 cells exposed to blue light. (**A**) The viability of ARPE-19 cells cultured in medium containing BLM serum was assessed using CCK-8 assay. (**B**) The viability of ARPE-19 cells cultured in medium containing BLM serum with or without blue light irradiation was tested using CCK-8 assay. (**C**) Western blot assay was used to examine NRF2 and HO-1 expression in ARPE-19 cells. ***p < 0.001 vs. Control group; ^##^*p* < 0.01, ^###^*p* < 0.001 vs. Model group.

Subsequently, to study the potential mechanism of BLM in blue light-induced retinal damage, the levels of proteins in NRF2/HO-1 pathway in retinal tissues was tested by western blot. It could be found that blue light irradiation markedly downregulated NRF2 and HO-1 expression in retinal tissues relative to the Control group (Fig. 4). Of note, both low and high doses of BLM intragastric administration elevated NRF2 and HO-1 expression, and more significant changes were found in the High group. ARPE-19 cells were maintained in DMEM containing various concentrations of BLM serum with or without of blue light irradiation. As displayed in Fig. 5A, medium supplemented with 5%-25% BLM serum had no significant effect on ARPE-19 cell viability. 15% serum (Low group), 20% serum (Middle group), and 25% serum (High group) were selected to perform the following assays. Relative to the Control group, blue light exposure notably attenuated ARPE-19 cell viability, which was elevated by BLM pretreatment (Fig. 5B). Moreover, we also discovered that NRF2 and HO-1 levels were markedly downregulated in the presence of blue light irradiation (Fig. 5C). On the contrary, medium containing BLM serum recovered NRF2 and HO-1 levels relative to the Model group. Together, these findings confirm that BLM activates NRF2/HO-1 signaling in rat retinal tissues and ARPE-19 cells challenged with blue light.

### BLM Alleviates Mitochondria Oxidative Stress and Senescence of Blue Light-Exposed ARPE-19 Cells by Activating NRF2/HO-1 Signaling

**Fig. 6:**
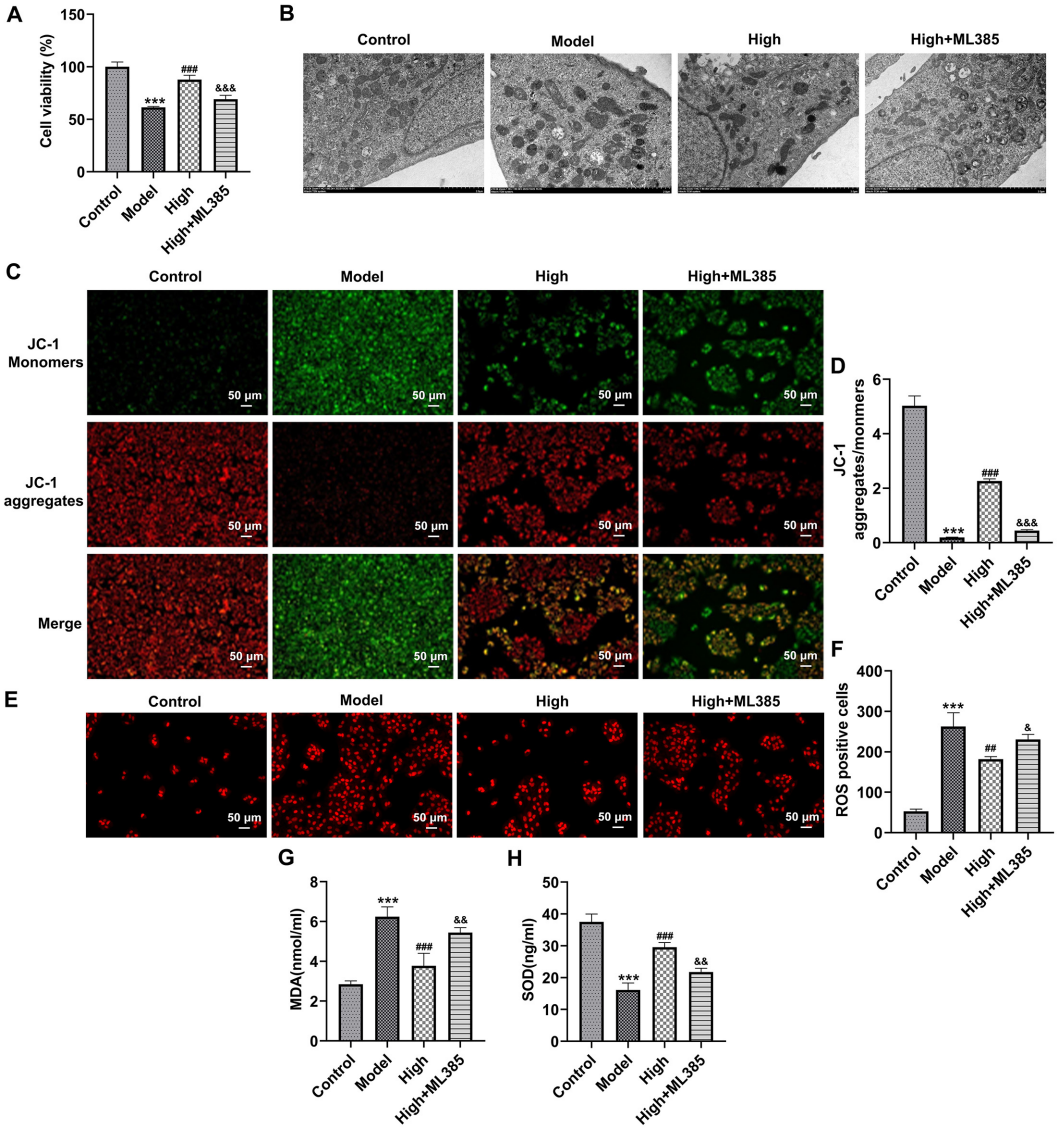
BLM alleviates the mitochondria oxidative stress of blue light-exposed ARPE-19 cells by activating NRF2/HO-1 signaling. (**A**) Cell viability was examined by CCK-8 assay. (**B**) Mitochondrial morphology was observed under a transmission electron microscope. (**C**) Mitochondrial membrane potential was analyzed using JC-1 staining. (**D**) Calculation of the ratio of JC-1 aggregates to JC-1 monomers. (**E**) Detection of ROS using DHE staining. (**F**) Quantification of DHE fluorescence intensity. Measurement of (**G**) MDA and (**H**) SOD levels using the corresponding commercial available kits. ***p < 0.001 vs. Control group; ^##^*p* < 0.01, ^###^*p* < 0.001 vs. Model group; ^&^*p* < 0.05, ^&&^*p* < 0.01, ^&&&^*p* < 0.001 vs. High group.

**Fig. 7:**
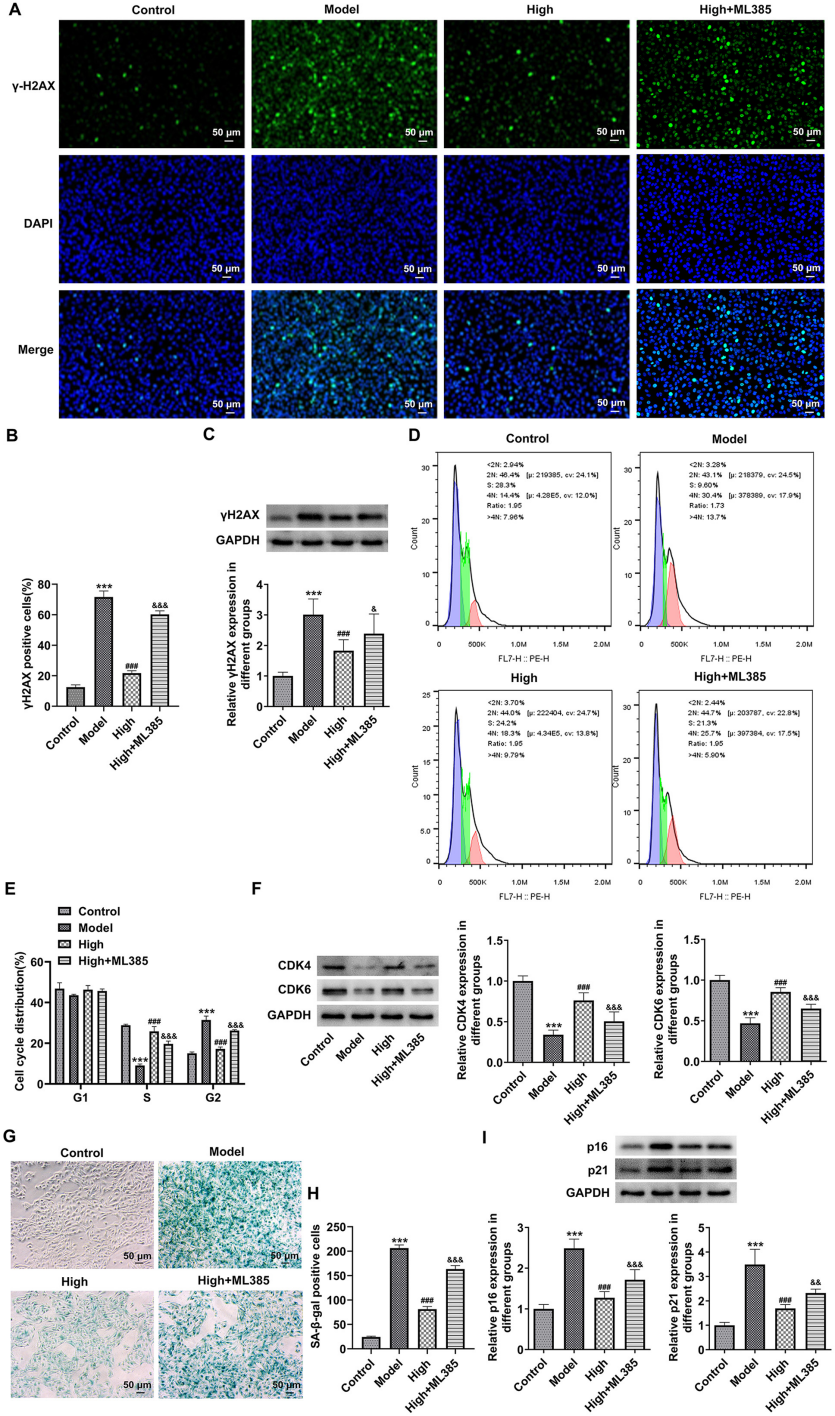
BLM alleviates the senescence of blue light-exposed ARPE-19 cells by activating NRF2/HO-1 signaling. (**A**) Immunofluorescence staining was used to analyze γH2AX expression. (**B**) Quantification of γH2AX positive area. (**C**) western blot assay was employed to measure γH2AX expression. (**D**) Cell cycle was examined with flow cytometry. (**E**) Quantification of cell cycle distribution. (**F**) Expression of CDK4 and CDK6 was detected using western blot. (**G**) Detection of cell senescence using SA-β-gal staining. (**H**) Quantification of γH2AX positive area. (**I**) p16 and p21 expression was examined by western blot. ***p < 0.001 vs. Control group; ^###^*p* < 0.001 vs. Model group; ^&^*p* < 0.05, ^&&^*p* < 0.01, ^&&&^*p* < 0.001 vs. High group.

To further clarify whether BLM improved blue light-triggered damage of the retina through NRF2/HO-1 signaling, ARPE-19 cells were treated with ML385, an NRF2 inhibitor. As what is observable from Fig. 6A, ML385 significantly decreased ARPE-19 cell viability as comparison to the High group. Additionally, the mitochondria of ARPE-19 cells suffered from blue light and exhibited serious injury with vacuole and cristae fracture (Fig. 6B). After BLM treatment, mitochondrial ridge breakage decreased and mitochondrial morphology recovered. The further NRF2 inhibitor intervention led to damaged mitochondrial morphology and increased cavitation compared with the High group. At the same time, when compared to the High group, the ratio of JC-1 aggregates fluorescence intensity to JC-1 monomers fluorescence intensity was remarkably decreased in ARPE-19 cells in the High+ML385 group (Fig. 6C and 6D). Besides, blue light exposure-induced increase of intracellular ROS, MDA levels, and decrease of SOD activity were markedly restored following BLM pretreatment (Fig. 6E-6H). However, ML385 reduced the impacts of BLM on intracellular ROS, MDA, and SOD levels in blue light-induced ARPE-19 cells. Concurrently, BLM conspicuously downregulated γH2AX level in ARPE-19 cells relative to the Model group, which was upregulated by ML385 intervention (Fig. 7A and 7B). Moreover, there was a notably drop in the percentage of ARPE-19 cells in S phase and elevated percentage of ARPE-19 cells in the G2 phase, coupled with downregulated expression of CDK4 and CDK6, were observed in the Model group (Fig. 7D-7F). As compassion to the Model group, ARPE-19 cells cultured in contained-BLM serum medium showed an elevated proportion of ARPE-19 cells in the S phase, decreased proportion of ARPE-19 cells in G2 phase and upregulated CDK4 and CDK6 expression. The further ML385 treatment alleviated the impacts of BLM on the cell distribution as well as CDK4 and CDK6 expression on ARPE-19 cells. Additionally, blue light promoted the senescence of ARPE-19 cells, accompanied by the elevated p16 and p21 levels (Fig. 7G-7I). The NRF2 inhibitor ML385 prevented blue light-triggered senescence of ARPE-19 cells from being caused by BLM. Altogether, BLM alleviates the mitochondria oxidative stress and senescence of blue light-treated ARPE-19 cells by activating NRF2/HO-1 pathway.

## Discussion

There has been a surge in scientific interest due to the potential health risks related to blue light exposure, especially the irreversible photochemical damage to eye tissue, has attracted increased research attention. This study demonstrated that BLM, an alcohol extract of marigold, could protect against blue light-triggered damage of rat retinal tissues and ARPE-19 cells, as evidenced by the improved histopathological changes, alleviated mitochondria oxidative stress and attenuated senescence of tissues and cells. More importantly, we found that BLM activated NRF2/HO-1 signaling, and NRF2 inhibitor ML385 significantly abolished the influences of BLM on blue light-treated ARPE-19 cells, suggesting that BLM ameliorated optical damage caused by blue light by activating NRF2/HO-1 signaling.

Mitochondria, the key organelles that produce ROS, have been considered as possible targets and initiators of blue light damage to the retina [19]. Retinal tissue is enriched in mitochondria, which give it a high exposure ability under blue light irradiation, and numerous endogenous chromophores in the retina possess a large amount of mitochondria capable of absorbing blue light and inducing photochemical effects [20]. Hence, overexposure of the retina to blue light is prone to the accumulation of ROS, which in turn affects the function of mitochondria in retina [21, 22]. Electrochemical potential is necessary to maintain the MMP, which is a critical component of mitochondrial function [23]. An increase in MMP refers to the elevated electron transport, which will generate more ROS as by-products [24, 25]. An growing body of studies have confirmed that blue-light-induced retinal photochemical damage is commonly related to lower MMP and increased ROS levels [26, 27]. MDA (the end product of lipid peroxidation) and SOD (an important antioxidant enzyme) levels are commonly regarded as the markers of oxidative stress [27]. Analogously with previous research, in this study, significantly disordered mitochondrial morphology, reduced ratio of JC-1 aggregates to JC-1 monomers, elevated MDA content and reduced SOD activity were observed in both blue light-induced rat retinal tissues and ARPE-19 cells [28, 29]. A considerable body of intervention trials have investigated the influences of lutein, zeaxanthin, and meso-zeaxanthin on eye health and diseases [30, 31]. Importantly, the abovementioned changes caused by blue light are conspicuously ameliorated by BLM treatment, especially in the high-dose BLM group.

As reported, blue light exposure induces the structural damage to retinal pigment epithelium cells, reduces cell viability, elevates ROS, and increases cell senescence [32]. Oxidative stress is considered to be main factor inducing cellular senescence [33]. The lifespan of flies maintained in 12 h of blue light-emitting diodes and 12 h of darkness per day is remarkably lower than that maintained in continuous darkness or white light where blue light was inhibited [34]. Trevor R Nash *et al* also confirmed that adult flies exposed to blue light for 12 h a day facilitates the aging phenotype, leading to retinal cell damage [34]. Additionally, blue light exposure results in decreased proliferation, increased oxidative stress, and activated DNA damage of ARPE-19 cells [35]. γH2AX, a marker of DNA double-strand breaks, has been discovered to be increased in retina exposed to blue light, as comparison to white light, suggesting the DNA damage induced by blue light [35]. SA-β-gal is a reliable marker for senescent cells [36]. P16 and p21 are two markers of cellular senescence, and p21 is mostly activated early in the senescence progression, while p16 keeps cells senescent. [37]. In accordance with previous investigations, this work clearly proved that light exposure induced the senescence of retinal tissues and ARPE-19 cells [35, 38]. The infliences of BLM on blue light-induced senescence of the retina had been explored in this study and the results revealed the anti-senescence effects of BLM, as evidenced by the downregulated γH2AX, p16, and p21 expression and the reduced level of SA-β-gal in retinal tissues and ARPE-19 cells.

NRF2 is a crucial regulator of the antioxidant response, serving as the main mechanism preventing oxidative stress by inducing the expression of various antioxidants, including HO-1 [39]. An existing study has shown that when comparing the RPE cells of aging mice to those of younger mice, NRF2 expression is downregulated, and this downregulation might be associated with an increase in basal oxidative stress with aging [40]. Strikingly, NRF2 expression is downregulated in ARPE-19 cells and mice retina tissue challenged with blue light, and drugs targeting NRF2 can be used as a protective strategy for photoreceptor cells in AMD and blue light-induced damage of retina [28, 41]. Consistently, the total NRF2 expression in ARPE-19 cells is significantly reduced at 6 h of blue light exposure [41]. Accordingly, this study showed that blue light stimulation decreased NRF2 and HO-1 levels in both rat retina and ARPE-19 cells, which were restored by BLM treatment. Mounting evidence supported that lutein, zeaxanthin, and meso-zeaxanthin can regulate NRF2 signaling in the retina [42, 43]. Specially, as reported, lutein not only serves as a direct antioxidant, but also activates NRF2 in ARPE-19 cells [44]. Lutein reverses high glucose-mediated downregulation of NRF2 expression in ARPE-19 cells [45]. By activating NRF2 and upregulating NRF2-mediated phase II enzymes, zeaxanthin enhances anti-oxidative capacity and prevents cell death in ARPE-19 cells and rat retina [46]. In obesity-induced rodent model, lutein and zeaxanthin isomers elevates nuclear NRF2 and HO-1 expression in retinal tissues [47]. Particularly, meso-zeaxanthin has protective effects on the retina and the ability to inhibit oxidative stress of retina in rats fed with high-fat diet, which were achieved by activating NRF2/HO-1 pathway [43]. Furthermore, the influences of BLM on blue light-triggered ARPE-19 cell damage was inhibited by the NRF2 inhibitor ML385, indicating that BLM alleviates the mitochondria oxidative stress and senescence of blue light-exposed ARPE-19 cells via activating NRF2/HO-1 pathway.

## Conclusion

In a word, this experiment demonstrates that BLM, an alcohol extract of magrigold, can provide a protection against blue light-induced damage of rat retinal tissues and human retinal pigment epithelial cells by activating NRF2/HO-1 signaling to attenuate mitochondria oxidative stress and senescence. Our results suggest that BLM may be used as a nutritional supplement to prevent blue-light-induced retinal damage.
